# A Single Amino Acid Substitution in Poliovirus Nonstructural Protein 2C^ATPase^ Causes Conditional Defects in Encapsidation and Uncoating

**DOI:** 10.1128/JVI.02877-15

**Published:** 2016-06-24

**Authors:** Emmanuel Asare, JoAnn Mugavero, Ping Jiang, Eckard Wimmer, Aniko V. Paul

**Affiliations:** Department of Molecular Genetics and Microbiology, Stony Brook University, Stony Brook, New York, USA

## Abstract

The specificity of encapsidation of C-cluster enteroviruses depends on an interaction between capsid proteins and nonstructural protein 2C^ATPase^. In particular, residue N_252_ of poliovirus 2C^ATPase^ interacts with VP3 of coxsackievirus A20, in the context of a chimeric virus. Poliovirus 2C^ATPase^ has important roles both in RNA replication and encapsidation. In this study, we searched for additional sites in 2C^ATPase^, near N_252_, that are required for encapsidation. Accordingly, segments adjacent to N_252_ were analyzed by combining triple and single alanine mutations to identify residues required for function. Two triple alanine mutants exhibited defects in RNA replication. The remaining two mutations, located in secondary structures in a predicted three-dimensional model of 2C^ATPase^, caused lethal growth phenotypes. Most single alanine mutants, derived from the lethal variants, were either quasi-infectious and yielded variants with wild-type (wt) or temperature-sensitive (*ts*) growth phenotypes or had a lethal growth phenotype due to defective RNA replication. The K_259_A mutation, mapping to an α helix in the predicted structure of 2C^ATPase^, resulted in a cold-sensitive virus. *In vivo* protein synthesis and virus production were strikingly delayed at 33°C relative to the wt, suggesting a defect in uncoating. Studies with a reporter virus indicated that this mutant is also defective in encapsidation at 33°C. Cell imaging confirmed a much-reduced production of K_259_A mature virus at 33°C relative to the wt. In conclusion, we have for the first time linked a cold-sensitive encapsidation defect in 2C^ATPase^ (K_259_A) to a subsequent delay in uncoating of the virus particle at 33°C during the next cycle of infection.

**IMPORTANCE** Enterovirus morphogenesis, which involves the encapsidation of newly made virion RNA, is a process still poorly understood. Elucidation of this process is important for future drug development for a large variety of diseases caused by these agents. We have previously shown that the specificity of encapsidation of poliovirus and of C-cluster coxsackieviruses, which are prototypes of enteroviruses, is dependent on an interaction of capsid proteins with the multifunctional nonstructural protein 2C^ATPase^. In this study, we have searched for residues in poliovirus 2C^ATPase^, near a presumed capsid-interacting site, important for encapsidation. An unusual cold-sensitive mutant of 2C^ATPase^ possessed a defect in encapsidation at 37°C and subsequently in uncoating during the next cycle of infection at 33°C. These studies not only reveal a new site in 2C^ATPase^ that is involved in encapsidation but also identify a link between encapsidation and uncoating.

## INTRODUCTION

**FIG 1:**
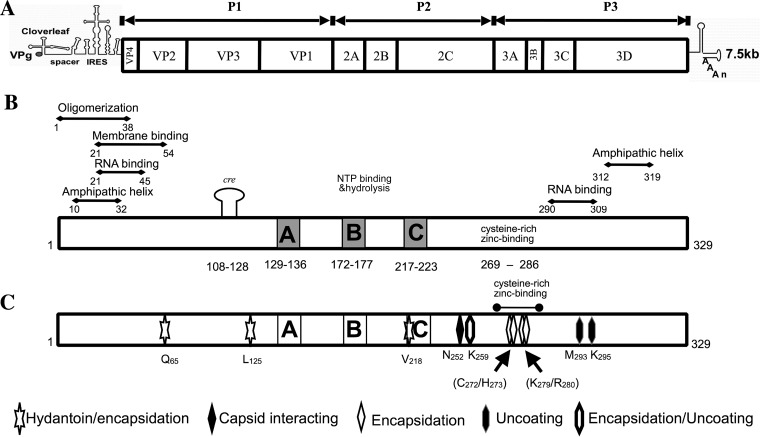
Poliovirus genome organization and functional motifs in the PV 2C^ATPase^ protein. (A) Poliovirus RNA contains a long 5′ nontranslated region (5′NTR), a single open reading reading frame, a short 3′NTR, and a poly(A) tail. (B) The locations of the known functional domains of the 2C^ATPase^ protein are illustrated. (C) Previously identified mutations in 2C^ATPase^ involved in encapsidation or uncoating are shown in detail. These include the hydantoin-resistant mutations (9), N_252_, the capsid-interacting site in the PV/CAV chimera (10), the residues involved in encapsidation derived from alanine scanning mutagenesis (12, 13), and the mutations leading to an uncoating defect in a mutant containing a nearby linker insertion (1).

Protein 2C^ATPase^ is a highly conserved nonstructural protein of the Picornaviridae, a family of plus-strand viruses that cause a wide range of important diseases in both humans and animals. 2C^ATPase^ maps roughly to the center of the polyprotein of poliovirus (PV) (Fig. 1A). Genetic, drug inhibition, and biochemical studies have identified multiple functions of this viral polypeptide, such as uncoating (1), host cell membrane rearrangement (2–4), RNA replication (5–8), and morphogenesis (9-13). During the last few years, we have concentrated our efforts on deciphering the role of 2C^ATPase^ in enterovirus assembly. We first provided evidence that the specificity of encapsidation is determined by an interaction between protein 2C^ATPase^ and capsid proteins rather than by an RNA encapsidation signal and RNA-protein interaction (10, 11). In a PV/coxsackievirus A20 (CAV20) chimera, the interaction site was identified to be between N_252_ of PV 2C^ATPase^ and E_180_ of CAV20 capsid protein VP3 (10). Subsequently, temperature-sensitive (*ts*) and quasi-infectious (*qi*) variants derived from alanine scanning mutagenesis of the PV 2C^ATPase^ polypeptide revealed other sites near the C terminus of the polypeptide that are involved in encapsidation (12, 13). In addition, suppressor mutations of one of these alanine mutants in 2C^ATPase^ mapped to capsid protein VP1 or VP3, confirming the importance of the 2C^ATPase^-capsid interactions for the formation of mature poliovirus particles (12). Encapsidation is difficult to study because this process is tightly linked with viral translation and RNA replication (14, 15). Additional conditional defective *ts* 2C^ATPase^ mutants would be useful in further enhancing our understanding of the role of this domain in this complex process.

Encapsidation is the last step in the viral replicative cycle, providing to newly synthesized genomes a protective protein coat that, in turn, is required for a virion's attachment to and penetration into a new host cell. Attachment and penetration lead to uncoating of the genome, a complex process involving structural alterations to the viral capsid and finally the release of infectious genomic RNA into the cytoplasm. With poliovirus, uncoating begins with the loss of VP4 from the capsid, followed by the loss of VP2 (Fig. 1), and finally the dissociation of VP1/VP3 and the viral RNA (16–20).

The RNA genome of PV is about 7,500 nucleotides (nt) long and encodes a polyprotein with one structural domain (P1) and two nonstructural domains (P2 and P3) (Fig. 1A). The polyprotein is processed into precursor and mature proteins by viral proteinases 3C^pro^/3CD^pro^ and 2A^pro^ (21–23). In poliovirus, 2C^ATPase^ is 329 amino acids long, and based on amino acid sequence analyses, it is classified as a member of the superfamily III helicases, which form hexameric ring structures (24). Such proteins contain three conserved motifs, two of which are typical nucleoside triphosphate (NTP)-binding motifs (A+B) and the third one (C), downstream of motif B, contains an invariant asparagine preceded by a stretch of hydrophobic residues, but its exact function is unknown (Fig. 1B). Downstream of motif C is residue N_252_, which is involved in the interaction with VP3 in a PV/CAV20 chimera (10). Poliovirus 2C^ATPase^ possesses ATPase activity *in vitro* (25–27), which is inhibited by guanidine hydrochloride (GnHCl) (27), a specific inhibitor of enterovirus RNA replication (28). Numerous attempts to discover helicase activity have failed in the past, although recently an RNA chaperone-type activity was reported to be associated with the 2C^ATPase^ protein of EoV, a picorna-like virus (29). Near its N terminus, the PV protein contains an amphipathic helix (7), an RNA binding domain (30), a membrane binding domain (31), and an oligomerization domain (Fig. 1B) (32). The central and C-terminal domains of the protein possess serpin (serine protease inhibitor) motifs, and accordingly 2C^ATPase^ inhibits the proteinase activity of 3C^pro^ both *in vitro* and *in vivo* (33). Near the C terminus, the protein contains another amphipathic helix (8) and a cysteine-rich Zn^2+^ binding domain (34). The protein has the ability to oligomerize through sequences near its N terminus (32) and to interact with viral proteins 2B/2BC, 3A/3AB, 3C^pro^, and VP3 (10, 33, 35, 36) and cellular protein reticulon 3 (37). The 2BC precursor of 2C^ATPase^ also interacts with cellular protein valosin-containing protein (VCP)/p97 (38). A small RNA hairpin *cre* in the coding sequence of 2C^ATPase^ (Fig. 1B) serves as the template for the uridylylation of VPg during RNA synthesis (39, 40), but the *cre* function is totally independent of the 2C^ATPase^ function.

The initial observation that linked 2C^ATPase^ to encapsidation came from experiments with hydantoin, a drug that inhibits virus growth at the stage of encapsidation (9). Drug-resistant variants were identified that mapped to the N-terminal and central portions of the polypeptide (Fig. 1C), but there was no clear correlation between these sites and other known motifs (9, 12). Early genetic studies also indicated the involvement of 2C^ATPase^ in encapsidation. A *ts* mutant with an insertion in 2C^ATPase^ yielded suppressor mutations that resulted in a cold-sensitive uncoating defect (1). This finding suggested that 2C^ATPase^ has a role in determining some aspect of virion structure. Our charged-to-alanine scanning mutagenesis, noted above, has revealed that residues K_279_ and R_280_ near the C terminus of the protein in a Zn^2+^ binding domain are also required for encapsidation (12). In addition, we identified a *ts* mutant (C_272_A H_273_A), also in the Zn^2+^ binding domain of the protein, defective in encapsidation (13). The delayed growth kinetics of this virus suggested the possibility of an additional uncoating defect.

In this study, we used alanine mutagenesis of amino acids near N_252_, the presumed 2C^ATPase^-VP3 interaction site within a variable segment of the 2C^ATPase^ polypeptide (10), with the aim of identifying additional residues in this domain that are involved in encapsidation. Most of these mutations introduced conservative amino acid changes with the replacement of hydrophobic residues with alanine. We started with triple alanine mutations, and from the lethal mutations, we selected single amino acid changes for further analyses. It should be noted such an approach, combining triple and single alanine scanning mutagenesis, had been used in the past successfully to identify essential residues in the NS5A protein of hepatitis C virus (HCV) (41). Using this approach, we identified two lethal triple alanine mutants and two others with *ts* or quasi-infectious growth phenotypes that were defective in RNA replication. Single residues when changed to alanine also conferred wild-type (wt)-like, lethal, *ts* or quasi-infectious growth phenotypes. The most interesting mutant (K_259_A) was cold sensitive in growth, and it possessed an encapsidation defect, which resulted in a severe delay in uncoating during the next cycle of virus infection.

## MATERIALS AND METHODS

### Plasmids.

Plasmid pT7PVM contains the full-length infectious cDNA of PV1(M). pT7R-Luc-PPP is an infectious Renilla luciferase reporter virus construct in which the 311-amino-acid-long R-Luc polypeptide is expressed as an N-terminal fusion of the PV polyprotein (10). pT7F-Luc PP is a replicon firefly luciferase (F-Luc) construct in which the structural P1 domain is replaced with F-Luc coding sequences in the pT7PVM background.

### Site-directed mutagenesis.

The standard site-directed mutagenesis protocol was used to obtain the desired mutations for both the triple alanine and single alanine mutants of PV 2C^ATPase^. In each case, the desired residues were replaced with alanine by changing the corresponding codons. An XhoI/HpaI fragment, spanning parts of the 2C^ATPase^ and 3A coding regions, was used as the template for site-directed mutagenesis. The mutated sites and corresponding codon changes are summarized in Tables 1 and 2. After sequencing analysis, the designed 2C^ATPase^ mutations were independently subcloned into pT7PVM or the Renilla luciferase reporter virus plasmid (R-Luc-PPP) for K_259_A.

**TABLE 1:** Summary of the growth phenotypes of triple alanine mutants surrounding N_252_, a capsid-interacting site in poliovirus 2C^ATPase^

Mutant	Mutations in 2C^ATPase^	Time of full CPE (growth phenotype) at^*a*^:		
		33°C	37°C	39.5°C
FMI/AAA	F_244_A, M_246_A, I_248_A			
QVM/AAA	Q_249_A, V_250_A, M_251_A	Transfection (*qi ts*)	Transfection (*qi ts*)	Passage 2 (*ts*)
EYS/AAA	E_253_A, Y_254_A, S_255_A	Transfection (*ts*)	Passage 2 (*ts*)	
GKL/AAA	G_258_A, K_259_A, L_260_A			

a *ts*, temperature sensitive; *qi*, quasi-infectious.

**TABLE 2:** Summary of the growth phenotypes of individual amino acid mutants of nonviable FMI/AAA and GKL/AAA mutants

Mutant	Nucleotide		Time of full CPE	Growth phenotype of alanine mutant^*b*^	Variant^*a*^	Growth phenotype of variant mutant^*b*^
	wt	Alanine mutant^*a*^				
F_244_A	TTC	gcC		Nonviable		
M_246_A	ATG	gcc	Transfection	Viable	gTc(valine)	*ts*
I_248_A	ATT	gcc	Transfection	*qi*	gTc(valine)	*ts*
K_259_A	AAA	gcA	Transfection	*cs*	gcA	*cs*
L_260_A	TTG	gca	Passage 1	*qi*	gTa(valine)	wt-like
G_258_A	GGG	Gcc	Transfection	Viable	Gcc	wt-like

a Lowercase letters indicate changed nucleotides.

b *ts*, temperature sensitive; *cs*, cold sensitive; *qi*, quasi-infectious.

### RNA transcription.

The wt and mutant plasmid DNAs of pT7PVM were linearized with EcoRI, and the pT7R-Luc-PPP plasmids were linearized with PvuII. The linearized plasmids were transcribed with T7 RNA polymerase.

### RNA transfections.

RNA transcripts (3 to 10 μg) were transfected into 35-mm-diameter HeLa R19 cell monolayers by the DEAE-dextran method, as previously described (42), and incubated at the indicated temperatures. Two days posttransfection at 37 and 39.5°C or 3 days after transfection at 33°C, viruses, if any, were harvested. Full cytopathic effect (CPE) was defined as the stage where 90% to 95% of the cells displayed CPE. Plates of monolayer cells that did not show signs of CPE were freeze-thawed three times and centrifuged to remove cell debris from the virus supernatant. Fresh monolayers were independently inoculated with these supernatants. Titers (PFU per milliliter) and phenotypes of all viable viruses that displayed CPE at 33°C were determined for viruses plaqued at 33, 37, and 39.5°C. The genotypes of viable viruses were confirmed by reverse transcription-PCR (RT-PCR) and sequencing analyses.

### *In vitro* translation.

Cytoplasmic extracts were prepared using HeLa S3 suspension cells, as described before (42). *In vitro* RNA translations were performed in the presence of Tran^35^S-label at 34°C overnight (42). Viral proteins were separated by SDS-PAGE (12.5% acrylamide), and the protein bands were visualized by autoradiography.

### Plaque assays.

Plaque assays were performed on HeLa R19 monolayers using 0.6% tragacanth gum. After 72 h of incubation at 33°C or 48 h of incubation at 37 or 39.5°C, the viral plaques were developed with 1% crystal violet (42).

### RT-PCR and sequencing analysis of isolated viral RNA from purified plaques.

Total RNA was extracted from 200 μl lysate with 800 μl TRIzol reagent (Invitrogen) and reverse transcribed into cDNA using SuperScript III reverse transcriptase (Invitrogen). PCR products were generated using high-fidelity Fusion polymerase (Finnzyme). PCR products were purified and further sequenced.

### qRT-PCR.

Viral RNA was quantitated by one-step real-time quantitative reverse transcription-PCR (qRT-PCR). HeLa cells were infected with wt PV or with viable triple alanine mutants (QVM/AVA and EYS/AAA) for 0, 2, 4, and 8 h. The infected cells were washed with phosphate-buffered saline (PBS), and total RNAs were extracted with TRIzol reagent (Invitrogen). Two hundred to 300 ng of total cellular RNAs was used to perform one-step qRT-PCR (Quanta Biosciences) with primers binding to the P1 structural region. GAPDH (glyceraldehyde-3-phosphate dehydrogenase) RNA was used as an internal control.

### Luciferase assays.

Monolayer HeLa R19 cells were independently transfected with 3 to 5 μg of R-Luc-PPP reporter virus transcript RNAs derived from cDNAs linearized with PvuI. The transfected cells were incubated at 33, 37, and 39.5°C overnight in Dulbecco's modified Eagle's medium (DMEM) with 2% (vol/vol) bovine calf serum (BCS) in the presence and absence of 2 mM GnHCl. Luciferase activity was determined on the cell supernatants after three freeze-thawing steps. Cell supernatant (100 μl) was mixed with 20 μl Renilla luciferase (R-Luc) assay reagent (Promega luciferase assay system; catalog no. E2810), and R-Luc activity was measured in an Optocom I luminometer (MGM Instruments, Inc.). Two hundred fifty microliters of cell supernatants from transfections in the absence of GnHCl was passaged once in the presence and absence of 2 mM GnHCl. Luciferase activity was determined in the supernatants after an overnight incubation. The R-Luc ratio was calculated as luciferase activity without GnHCl (−GnHCl) divided by luciferase activity with GnHCl (+GnHCl) in either transfection or infection.

### Immunofluorescence cell imaging.

HeLa R19 cells were infected with wt or K_259_A virus at a multiplicity of infection (MOI) of 5 and incubated at 37°C for 4 h, 35°C for 5 h, or 33°C for 6 h. Cells were fixed with 4% paraformaldehyde for 15 min at room temperature. Then the cells were permeabilized with 0.2% saponin and probed for mature virus with A12 primary antibody, which exclusively recognizes PV mature virus (43), and secondary Alexa Fluor 488-conjugated antibody. The localization of 2C^ATPase^ was determined in the same cell using monoclonal 2C^ATPase^ antibody and an Alexa Fluor 555-conjugated secondary antibody. Images were taken by Nikon's superresolution three-dimensional (3D)-structured illumination microscopy (SIM).

### Transmission electron microscopy.

PV 2C wt and K_259_A viruses, grown at 37°C on HeLa R19 cells, were purified by CsCl density gradients. The recovered viruses were dialyzed in PBS overnight and were fixed in 5% glutaraldehyde grade 1 solution for 5 min. Fixed viruses were transferred to an imaging grid and stained with uranyl acetate. Viruses were visualized with a transmission electron microscope (FEI Tecnai).

### Western blot analyses.

Monolayer HeLa R19 cells were infected at an MOI of 5 with wt or K_259_A viruses and incubated at 33, 37, and 39.5°C. The growth media were aspirated at 3, 5, 6.5, and 8 h, respectively, and lysed with detergent. Cell lysates from the various time points were run on an SDS-PAGE gel and transferred to a nitrocellulose membrane. 2C^ATPase^ protein and VP3 (capsid protein) were detected using primary mouse monoclonal and rabbit polyclonal antibodies, respectively.

## RESULTS

**FIG 2:**
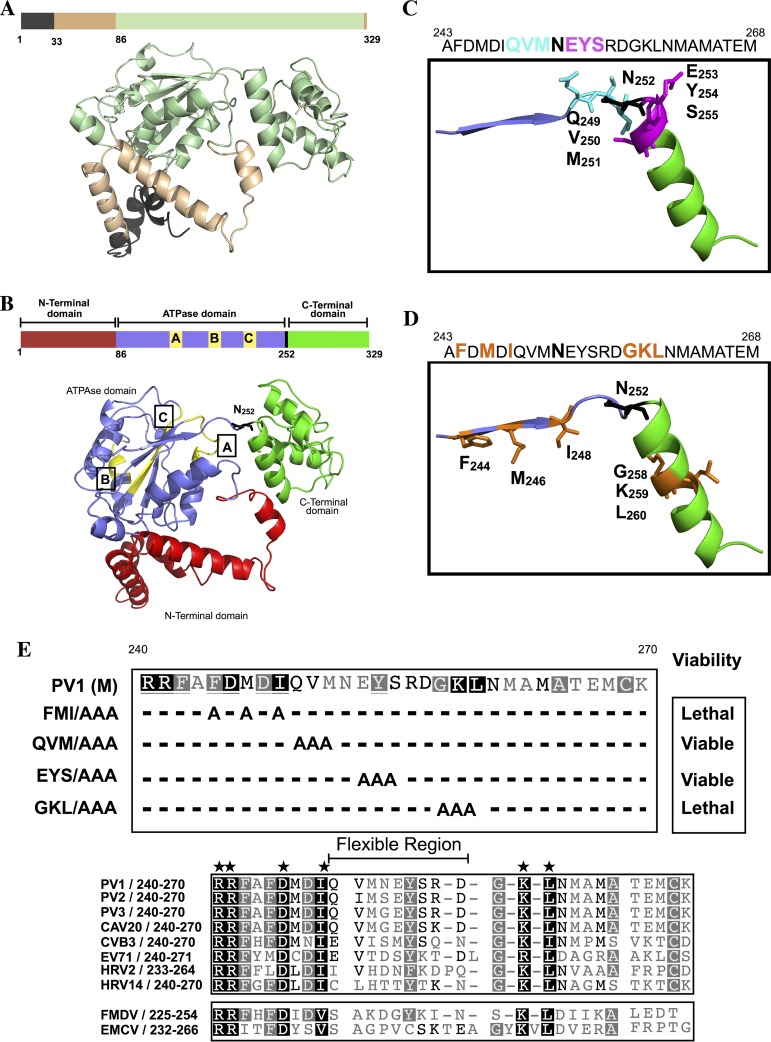
Phyre2-predicted model of the PV 2C^ATPase^ polypeptide structure and the locations of the FMI/AAA and GKL/AAA mutations on the structure. (A) The amino acid sequence of PV1(M) 2C^ATPase^ was submitted to the Phyre2 online server to generate a 3D structural model (Materials and Methods). The structure of the N-terminal domain, highlighted in black, is highly unreliable. Sequences highlighted in gold are predicted with good confidence. The 2C^ATPase^ structure shown in light green is predicted with 100% confidence. (B) The predicted three domains in the 2C^ATPase^ structure. The N-terminal domain (red) consists of helical structures, the central helicase domain (blue) contains the NTP binding/helicase boxes A, B, and C, and the C-terminal domain (green) consist mostly of helical structures. The flexible region between the central and C-terminal domains contains residue N_252_. (C and D) Locations of the triple alanine mutations on the predicted 2C^ATPase^ structure are illustrated. The QVM residues are in a flexible domain, while the EYS residues are partly in an α-helical structure. The FMI residues are predicted to be located in a β sheet, and the GKL residues are in an α-helix structure. (E) Alignment of picornavirus 2C^ATPase^ proteins surrounding residues N_252_. (Top) The locations of the triple alanine mutants in the 2C^ATPase^ polypeptide are shown. The growth phenotypes of the mutants are also indicated. (Below) The amino acid sequence of picornavirus 2C^ATPase^ proteins, surrounding N_252_, is shown. The highly conserved residues are indicated by stars over a black background, and the less conserved residues are shown with a gray background. Dashes indicate the absence of certain residues in the sequences alignment. The size and location of the flexible region are also indicated. The enterovirus 2C^ATPase^ proteins are boxed to separate them from the more distantly related picornaviruses (FMDV, Aphtovirus genus; EMCV [encephalomyocarditis virus], Cardiovirus genus).

Studies of the role of 2C^ATPase^ in PV encapsidation have been hindered by the multifunctional nature of the protein (see reference 12 and references therein) as well as by the stringent dependence in *cis* of translation > RNA replication > assembly (12, 14, 15, 42). Since 2C^ATPase^ plays an essential role in RNA replication, a step prior to particle assembly, mutations in 2C^ATPase^ leading to RNA replication defects will also prevent proper encapsidation. Our previous genetic studies with a CAV20/PV chimera indicated the importance of residue N_252_ in PV 2C^ATPase^ for an interaction with CAV20 capsid protein VP3, an interaction required for encapsidation (10). Unexpectedly, in the context of the PV polyprotein, N_252_ is not important for encapsidation because its replacement with A_252_, G_252_, S_252_, or D_252_ had no effect on virus growth (data not shown). We, therefore, extended the genetic analysis of assembly by introducing multiple mutations into the region surrounding the flexible domain harboring N_252_ (Fig. 2B and E). We speculate that in the PV background one or more residues in the vicinity of N_252_, in or near the flexible domain, might have a similar function in encapsidation as N_252_ in the context of the CAV20/PV chimera. In this study, we used alanine mutagenesis of selected residues to try to answer this question.

### 2C^ATPase^ three-dimensional structure prediction.

The propensity of PV 2C^ATPase^ to bind to membranes has thus far prevented the purification of the polypeptide in a soluble form in quantities sufficient to determine its three-dimensional structure. Only the glutathione *S*-transferase (GST)- and maltose binding protein (MBP)-tagged full-length PV 2C^ATPase^ polypeptides or the protein anchored to small nanodisk membrane bilayers have been obtained in soluble forms (27, 32, 44). In an attempt to obtain information about the location of the mutations that we have selected for analysis in the structure of the PV 2C^ATPase^ polypeptide, we used Phyre2, a protein homology/analogy recognition engine (45). The 2C^ATPase^ structure was modeled after chain C of the cellular transport protein VCP/p97 in a complex with ADP (46). VCP/p97 is a cellular ATPase that belongs to the class I AAA+ family (47). The server predicted a 2C^ATPase^ structure for 92% of the residues with greater than 90% confidence in the modeled polypeptide (Fig. 2A). The N-terminal segment, highlighted in black, is highly unreliable, while sequences highlighted in light brown can be predicted with good confidence (Fig. 2A). The remainder of the sequence, shown in light green, is predicted with 100% confidence.

The predicted 2C^ATPase^ model can be subdivided into three structured domains (N-terminal, central helicase, and C-terminal) connected by flexible regions (Fig. 2B). The N-terminal domain (red) contains helical regions. It should be noted that within this region the removal of the amphipathic helix (residues 1 to 33), which affects membrane binding, was found to be sufficient for the production of truncated soluble foot-and-mouth disease virus (FMDV) 2C^ATPase^ protein (48). The central domain (blue) contains the conserved NTP binding boxes (yellow) A, box B of all helicases, and box C, specific for SF3 helicases (24). Phyre2 predicted the structure of this domain with 100% confidence to contain both α helices and β-sheet secondary structures. Residue N_252_ is located in a flexible region of the polypeptide between the central ATPase/helicase and C-terminal (green) domains of the polypeptide (Fig. 2B). The C-terminal domain contains the cysteine rich Zn^2+^ binding domain that is already known to be involved in encapsidation (12, 13).

In the flexible domain of the polypeptide, the amino acid sequences are not conserved among picornavirus 2C^ATPase^ proteins (Fig. 2E). This has led us to conclude that the role of N_252_ residue in C-cluster enterovirus assembly (1) is unique for the chimeric polyprotein consisting of CAV20 capsid and the poliovirus P2 domain. We speculate that in the poliovirus background, one or more residues in the vicinity of N_252_, in or near the flexible domain, might have a function similar to N_252_ in encapsidation.

### Construction of triple alanine mutants near residue N_252_ and their growth phenotypes.

**FIG 3:**
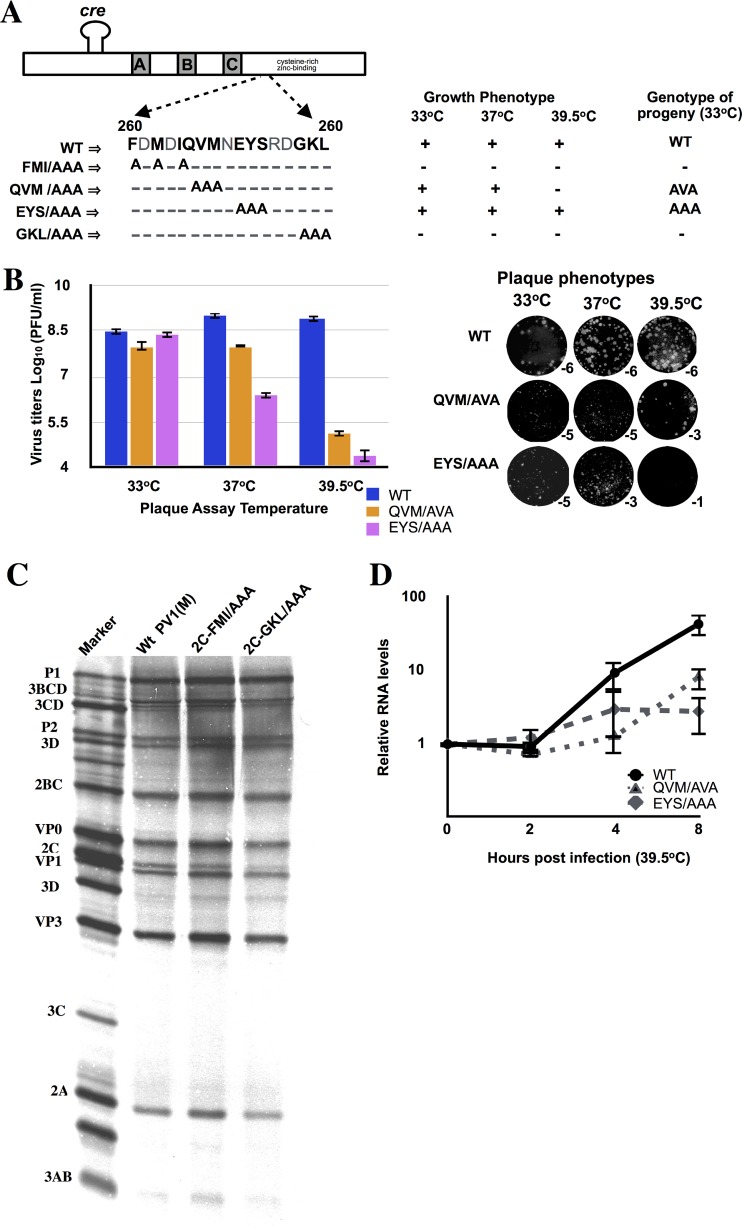
Characterization of PV 2C^ATPase^ triple alanine mutants. (A) Growth properties of mutants at different temperatures. The locations of N_252_ and of the triple alanine mutants on the 2C^ATPase^ sequence are illustrated on top. RNA transcripts of wt and mutant constructs were transfected into HeLa R19 monolayers and incubated at 33, 37, and 39.5°C for 48 h or until CPE (Materials and Methods). Freeze-thawed supernatants were used for passage on fresh HeLa R19 monolayers. The genotypes of recovered progeny viruses from 33°C passages are indicated. (B) Virus titers and plaque phenotypes of viable mutants QVM/AVA and EYS/AAA. Viruses derived from 33°C transfections were plaqued at 33, 37, and 39.5°C, and the titers were determined (Materials and Methods). The plaque phenotypes of the viruses at different temperatures are shown at the indicated dilutions. The lysates derived from the 33°C transfections were passaged 10 times, and the sequences of the full-length genomes of the progeny were determined. No additional genetic changes were observed. (C) *In vitro* translation of wt and mutant transcript RNAs. Transcript RNAs of the wt and of the lethal mutants were translated in HeLa cell extracts (Materials and Methods). (D) RNA levels in HeLa cells infected with mutant EYS/AAA and variant QVM/AVA. HeLa cells were infected at an MOI of 5 with wt and mutant viruses obtained from 33°C transfections, and the titer was determined at 37°C. The infected cells were harvested at various time points after infection, and total RNA was isolated from the lysates. RNA levels were determined by qPCR, as described in Materials and Methods.

Since we targeted all clusters of charged residues in 2C^ATPase^ for alanine mutagenesis in our previous work (12), in this study we selected triplet hydrophobic amino acids or single charged residues near N_252_ for analysis that have not yet been previously analyzed. We designed and constructed four triple alanine mutants—two just upstream of N_252_ (F_244_A M_246_A I_248_A [FMI/AAA] and Q_249_A V_250_A M_251_A [QVM/AAA]) and two just downstream of N_252_ (E_253_A Y_254_A S_255_A [EYS/AAA] and G_258_A K_259_A L_260_A [GKL/AAA]), respectively (Table 1; Fig. 3A). The triple alanine mutant FMI/AAA is predicted to be located in a β sheet, with QVM/AAA in the flexible region just upstream of N_252_ (Fig. 2C and D). The domain downstream of N_252_ is predicted to contain the other two triple mutants, EYS/AAA and GKL/AAA, which are either partly or fully within a helical region (Fig. 2C and D).

To compare the growth phenotypes of the triple alanine mutants with that of the wt virus, we transfected RNA transcripts into HeLa R19 cells. The transfected cells were incubated at 33, 37, or 39.5°C for about 72 h or until full cytopathic effect (CPE) developed. Two constructs (FMI/AAA and GKL/AAA) produced no progeny even after 10 passages on fresh HeLa cells at all 3 temperatures (Table 1; Fig. 2E and 3A). One mutant (EYS/AAA) exhibited wt-like growth at 33°C, but at 37°C progeny was produced only after two passages on fresh HeLa cells (Table 1; Fig. 3A). The second viable mutant (QVM/AAA) was defective in growth at 39.5°C but produced progeny at 33 or 37°C (Table 1; Fig. 3A). The titers of lysates of the two viable viruses, obtained from 33°C transfections, were determined by plaque assays at 33, 37, or 39.5°C. As shown in Fig. 3B, virus titers at 37 and 39.5°C were significantly lower with both mutants than what was obtained with the wt virus. The QVM/AVA variant and the EYS/AAA virus exhibited mostly tiny plaques at all temperatures tested (Fig. 3B).

Viral RNAs were extracted from lysates of the two viable triple alanine mutants grown at different temperatures and were subjected to RT-PCR and full-length genome sequencing. The results indicated that QVM/AAA is quasi-infectious (49): progeny viruses isolated from HeLa cell lysates were always found to be genetic variants. The variant produced had a single nucleotide reversion QVM/AVA. No nucleotide substitutions were observed with the EYS/AAA mutant (Fig. 3A).

### Nonviable triple alanine mutants exhibit normal protein synthesis.

To rule out the possibility that the lethal growth phenotypes of the FMI/AAA and GKL/AAA mutants were due to a defect in protein translation or polyprotein processing, we translated RNA transcripts of these mutants in HeLa cell extracts (42). After incubation for 8 h at 34°C, the samples were analyzed by SDS-PAGE. As shown in Fig. 3C, both mutants exhibited normal protein synthesis and polyprotein processing profiles. Surprisingly, the amino acid substitutions with alanine did not influence the migration of the 2C^ATPase^-related polypeptides compared to wt translation patterns.

### Triple alanine *ts* mutants are defective in RNA replication at 39.5°C.

The two *ts* triple mutants EYS/AAA and the partial revertant of QVM/AAA (QVM/AVA) were further analyzed to test for defects in RNA replication. Viruses grown at 33°C were used to infect HeLa cells at 39.5°C, and aliquots were taken at various times postinfection. Plus-strand RNA levels in lysates of cells infected with these viruses were measured by qPCR. Both mutants exhibited a severe defect in RNA replication at 39.5°C compared to the wt virus (Fig. 3D). Since encapsidation is dependent upon RNA replication (15, 42), these mutants were not further analyzed for any additional encapsidation defects.

### Construction and growth properties of single alanine mutants.

**FIG 4:**
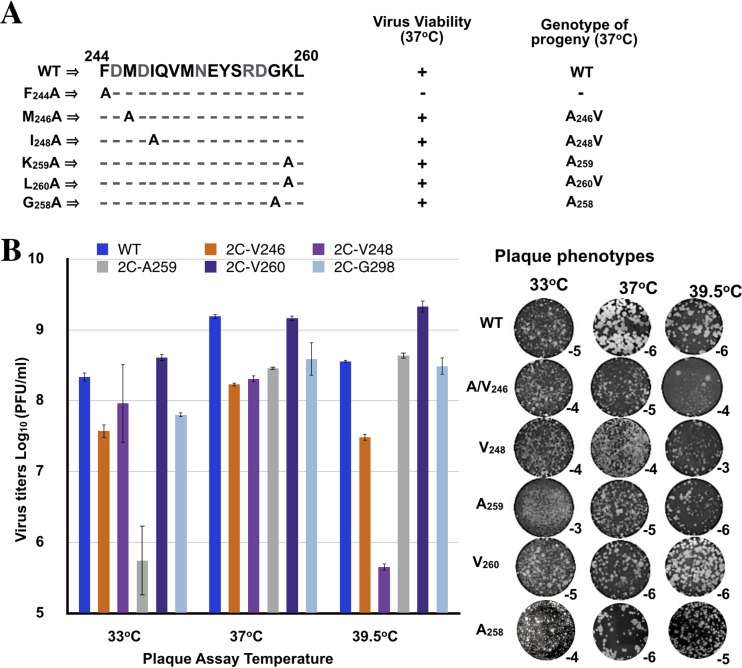
Growth phenotypes of single alanine mutants. (A) Virus viability and the genotype of progeny at 37°C. Single alanine mutants derived from the two nonviable triple amino acid mutants FMI/AAA and GKL/AAA were generated. They were tested for viability at 37°C, and the genotypes of the progeny were determined. (B) Virus titers and plaque phenotypes of single alanine mutants. Titers of viruses grown at 37°C were determined at 33, 37, and 39.5°C by plaque assay (Materials and Methods). The plaque phenotypes are shown on the right panel. Subscripts indicate the virus dilution at which the image was taken. It should be noted that the mixed plaque sizes seen with the lysate derived from M_246_A (39.5°C) presumably contain a mixture of the original alanine mutant (small) and of the V variant (large). Lysates derived from 37°C transfections were passaged 4 times, and the sequences of the full-length genomes were determined. No additional genetic changes were observed.

Triple alanine mutants that displayed a lethal growth phenotype (FMI/AAA and GKL/AAA) were subsequently scanned by single alanine mutagenesis to identify the specific residues responsible for the growth defect. Of the amino acids contained within FMI and GKL, six (F_244_A, M_246_A, I_248_A, G_258_A, K_259_A, and L_260_A) were mutated to alanine (Fig. 4A; Table 2). RNA transcripts of the six alanine mutant cDNAs were transfected into HeLa cells at 37°C, and their growth phenotypes were examined. Only one of the mutants (G_258_A) grew like the wt virus. The F_244_A mutant was not viable and did not produce any progeny after several passages on HeLa cells. The lethal growth phenotype was not due to a defect in translation or protein processing as shown by *in vitro* translation of mutant RNA transcripts in HeLa cell extracts (data not shown). qPCR analysis of viral RNA levels in infected cells indicated a defect in RNA replication (data not shown), and therefore the mutant was not further analyzed.

Two of the mutants (I_248_A and L_260_A) were quasi-infectious and produced A→V variants during transfection or first passage, an observation indicating that the original alanine residues at these positions were nonfunctional (Fig. 4A; Table 2). Although the M_246_A virus was viable, it mutated to a V during transfection, yielding an M_246_V variant. Titers of viruses derived from 37°C transfections were determined by plaque assay at 33, 37, and 39.5°C (Fig. 4B). The I_248_V variant was *ts* with a particularly strong growth defect at 39.5°C but less prominent at 33 or 37°C. The M_246_V variant exhibited mildly lower titer than the wt at all three temperatures tested, while the L_260_V variant grew nearly as well as the wt virus. The plaque sizes of the single alanine mutants or the valine variants were somewhat smaller at 37 or 39.5°C than that of the wt (Fig. 4B).

The last of the single mutants, K_259_A, produced progeny during transfection, and our preliminary studies indicated that it was cold sensitive. Importantly, it retained its original alanine mutation genotype even after several passages at 33°C (Fig. 4A and B). It should be noted that from the only two other cold-sensitive mutants of poliovirus known so far, one had a mutation in viral protein 3A with a defect in viral RNA synthesis (50), and the other was shown to be defective in uncoating due to a possibly defect in virion structure (1). This uncoating cold-sensitive mutant contained a linker insertion (4 amino acids) between residues 255 and 256, just upstream of K_259_, and two secondary mutations at M_293_V and R_295_K that were obtained after passaging at 39.5°C (1). Notably, a single N_140_S change was able to suppress the cold-sensitive growth phenotype, suggesting an interaction between the C-terminal domain of the polypeptide and a domain between boxes A and B of the NTP binding domain. We have similarly postulated an interaction between these domains from our previous alanine scanning analyses (12, 13). Based on these previous genetic studies with a linker insertion in 2C^ATPase^, our K_259_A mutant appeared to be a good candidate in the search for a possible encapsidation/uncoating defect.

### The PV 2C^ATPase^ K_259_A mutant exhibits a delay in growth and protein synthesis at 35 and 33°C.

**FIG 5:**
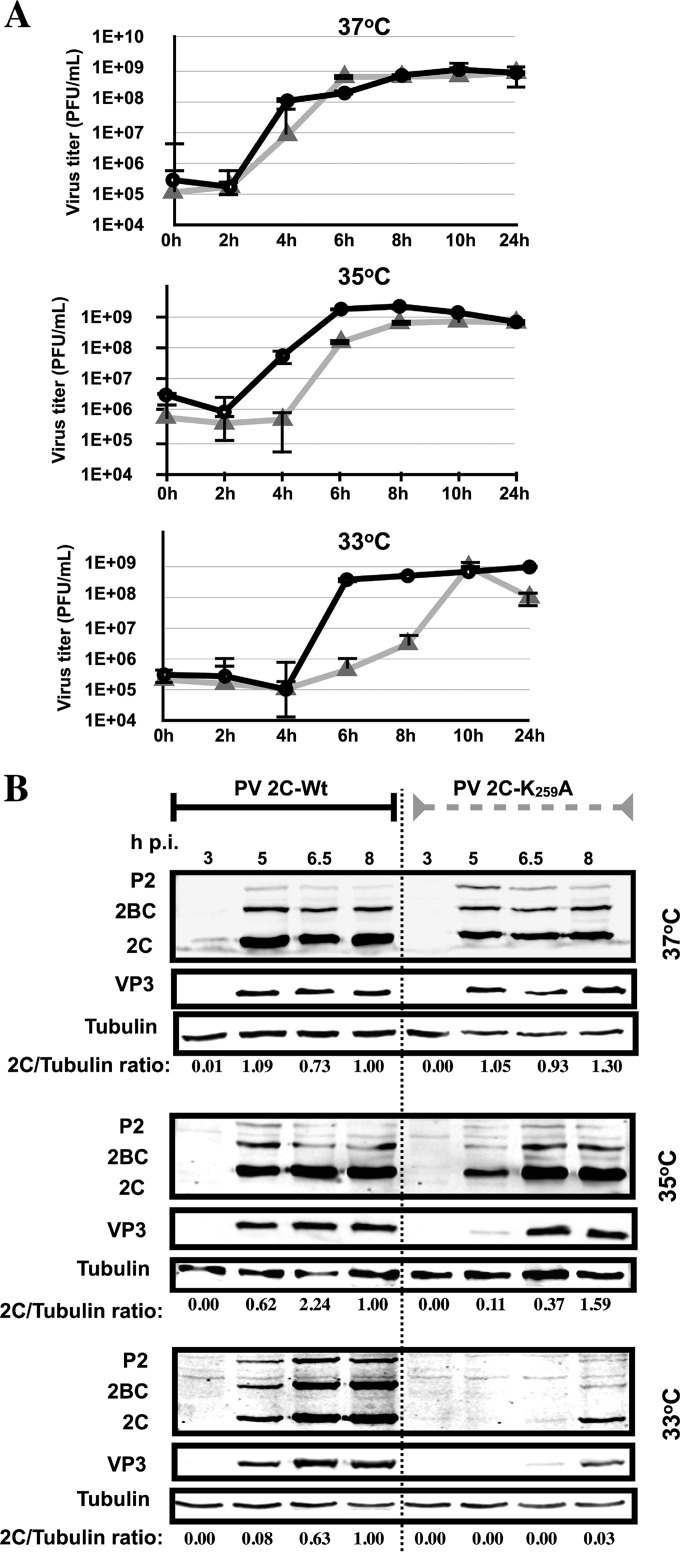
The 2C^ATPase^ K_259_A mutant is delayed in virus production and protein synthesis at the restrictive temperatures (35 and 33°C). (A) Growth curves of wt and K_259_A mutant polioviruses at the permissive (37°C) and restrictive (35 and 33°C) temperatures. HeLa cells were infected at an MOI of 5 with viruses derived from 37°C transfections. The titer of the viral progeny was determined by plaque assay at different times postinfection (Materials and Methods). Black lines indicate growth curves at 37°C, and gray lines indicate growth curves at 33°C. (B) Protein synthesis by the wt and K_259_A mutant measured by Western analysis. HeLa cells were infected at either 33, 35, or 37°C at an MOI of 5 with viruses derived from 37°C transfections. The infected cells were isolated at various times postinfection and lysed. The level of 2C^ATPase^-related proteins and of capsid protein VP3 were measured by Western analysis using a monoclonal antibody to 2C^ATPase^ and a polyclonal antibody to VP3, respectively, as described in Materials and Methods. Tubulin was used as a loading control. The experiment was carried out three times.

To learn more about the cold-sensitive phenotype of this mutant, viruses grown at 37°C were used to infect HeLa cells (MOI of 5) at 37, 35, or 33°C. Compared to the wt virus, the cold-sensitive mutant exhibited increasingly longer delays in growth when the temperature of the infection was reduced from 37°C to 35°C and then to 33°C, respectively (Fig. 5A). It took 10 h postinfection for the virus titer of PV 2C^ATPase^ K_259_A to catch up with the titer produced by the wt virus at 6 h postinfection (Fig. 5A). These results suggested a defect at an early step of infection with the PV 2C^ATPase^ K_259_A virus, possibly in uncoating.

**FIG 6:**
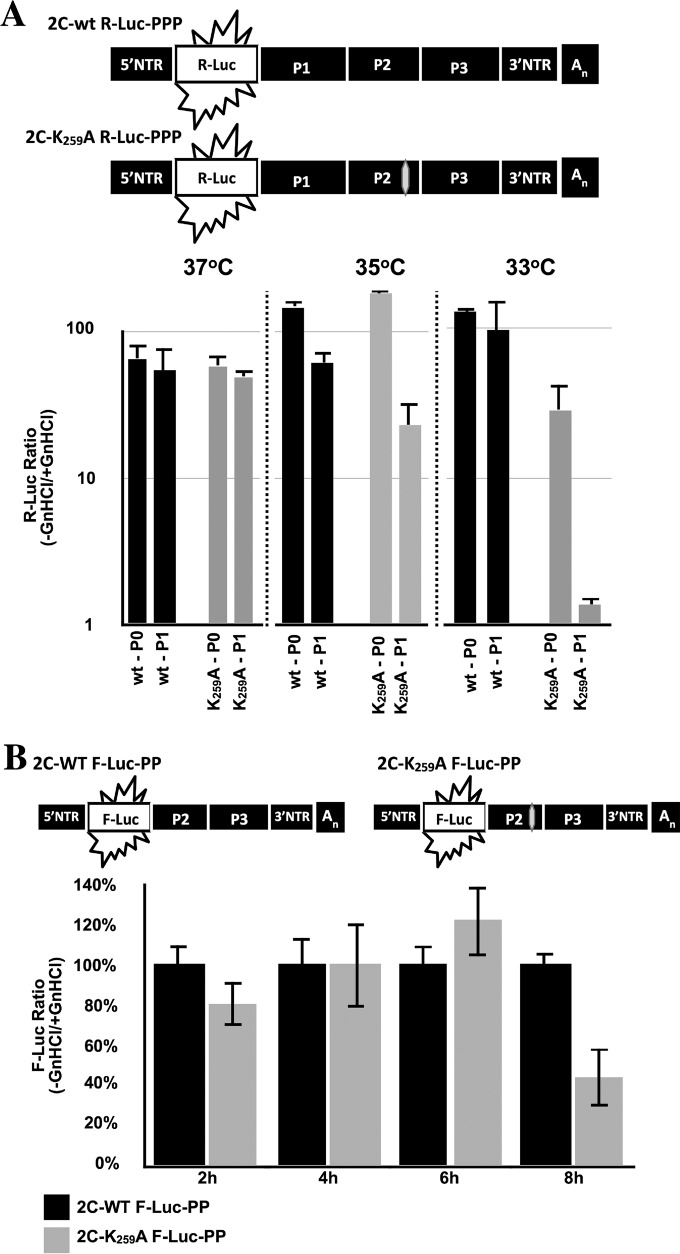
The K_259_A 2C^ATPase^ mutant possesses an encapsidation defect at 33°C. (A) Genome structure of a Renilla luciferase (R-Luc) reporter virus (R-Luc-PPP). The R-Luc gene was fused between the 5′NTR and P1 structural proteins, flanked by a 3CD^pro^ cleavage site. Wild-type and mutant K_259_A 2C^ATPase^ reporter virus RNA transcripts were transfected into HeLa cells at 33 or 37°C, in both the absence and presence of GnHCl (Materials and Methods). R-Luc assays were performed at 8 h posttransfection. Aliquots of the lysates from the transfections were used to infect fresh HeLa cells, in both the absence and presence of GnHCl. R-Luc assays were performed at 8 h postinfection. R-Luc ratios were calculated by dividing the raw R-Luc values in the absence by the R-Luc values in the presence of GnHCl. (B) The genome structure of firefly luciferase PV1(M) replicons used in the experiment is shown above. A time course of RNA replication was measured with the wt and the mutant 2C^ATPase^ K_259_A mutant using F-Luc replicons. Monolayer HeLa R19 cells were transfected with 3 to 5 μg of firefly Renilla luciferase replicon transcript RNAs. Transfected cells were incubated for 2, 4, 6, and 8 h at 33°C in the presence or absence of 2 mM GnHCl. Luciferase activity was determined on the cell supernatants after three freeze-thawing steps. F-Luc activity with the wt virus is taken as 100%. The experiment was carried out three times.

Since in the viral life cycle protein synthesis follows immediately after uncoating, we reasoned that the K_259_A mutant might also exhibit a delay in protein synthesis at 33 and 35°C compared to the wt virus. Virus grown at 37°C was used to infect HeLa cells at different temperatures at an MOI of 5 (Fig. 5B). At various times postinfection, lysates were analyzed on SDS-polyacrylamide gels, and the 2C^ATPase^-related proteins were identified by Western analysis using an antibody to 2C^ATPase^. Relative to the wt virus, there was a small delay in protein synthesis by the mutant at 37°C, and there were increasingly longer delays when the temperature was reduced from 37°C to 35 and 33°C, respectively (Fig. 5B). The results parallel the growth kinetics of the mutant virus at 33, 35, and 37°C compared to the wt virus and provide support for our hypothesis that the cold-sensitive mutant is defective in uncoating at the restrictive temperatures. In agreement with these experiments, we observed no difference in the kinetics of RNA replication in experiments where RNA transfection, instead of infection, initiated the replication cycle of an F-Luc replicon at 33°C (Fig. 6B).

### The PV 2C^ATPase^ K259A mutant is defective in encapsidation at 33 and 35°C.

The delayed growth and protein synthesis by the K_259_A mutant at 33°C suggested the possibility of a defect at some stage of uncoating. However, since 2C^ATPase^ is not a part of the virus particle and is synthesized after viral entry and uncoating, a direct role for this protein in this process can be ruled out. To decipher the mechanism by which a mutation in 2C^ATPase^ might affect uncoating, as we have hypothesized, we tested for defects in RNA replication and encapsidation using an R-Luc reporter virus (Fig. 6A). In this construct, R-Luc is fused to the N terminus of the poliovirus polyprotein (10). After infection, the chimera synthesizes the polyprotein from which the N-terminal R-Luc reporter protein is cleaved by 3CD^pro^, after which it signals the extent of protein translation and RNA replication (10). We used T7 RNA transcripts of the chimeric virus constructs (wt and K_259_A mutant) and transfected these into HeLa R19 cells at different temperatures in the absence and presence of guanidine hydrochloride (GnHCl), a potent inhibitor of PV RNA replication (28). Luciferase activity was measured 16 h posttransfection. In the presence of GnHCl, the R-Luc activity measures translation of the input RNA, while in the absence of the drug, the R-Luc signal represents both translation and RNA replication. To gauge encapsidation, cell lysates from transfections made in the absence of GnHCl were then passaged to fresh HeLa cells, and R-Luc activity was measured 8 h postinfection. Only virions that were assembled during the first incubation will be able to infect the second set of HeLa cells. The results indicate that at 37°C, both RNA replication and encapsidation of the K_259_A mutant nearly matched the level observed with the wt construct (Fig. 6A). At 35°C, passaging to new HeLa cells reduced the R-Luc signal for both the wt and mutant constructs, although the decrease was more pronounced with the mutant, an observation suggesting a small encapsidation defect relative to the wt. However, at 33°C, the mutant exhibited a small (∼2-fold) reduction in RNA replication but a striking 25-fold decrease in R-Luc signal that measures encapsidation. We consider it highly unlikely that such a small decrease in RNA replication would result in such a very large defect in encapsidation. Therefore, we conclude that the K_259_A mutant is severely defective in encapsidation at 33°C.

We also carried out a more detailed analysis of the kinetics of RNA replication with the wt and K_259_A mutant using F-Luc replicons (Fig. 6B). The RNA of the replicons was directly transfected into HeLa cells at 33°C, and F-Luc activity was measured at the indicated time points. Again, the experiment was carried out with or without the GnHCl inhibitor. Under these conditions, where uncoating is not involved, the kinetics of early RNA replication by the wt and mutant were essentially identical. Only at 8 h posttransfection did we observe a 2-fold reduction in RNA levels, confirming the results obtained by the R-Luc experiments (Fig. 6).

### Immunofluorescence imaging shows inhibition of mature virus production with the K_259_A mutant at 33°C.

**FIG 7:**
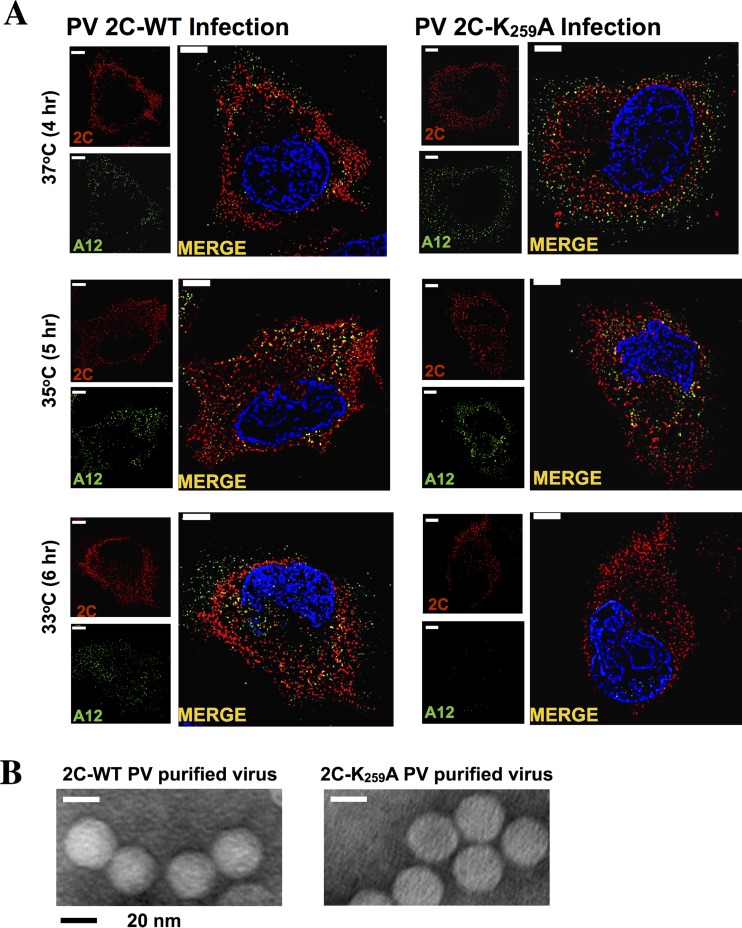
Immunofluorescence imaging of PV 2C wt and PV 2C K_259_A mutant virus-infected HeLa cells. HeLa cells were infected with wt or PV 2C K_259_A virus at an MOI of 5. Cells were incubated for 4 (37°C), 5 (35°C), or 6 (33°C) h and were fixed with paraformaldehyde. Infected cells were probed with primary antibody against 2C^ATPase^ and mature virus (monoclonal antibody A12), followed by Alexa Fluor 555 (red)- and 488 (green)-conjugated antibodies, respectively. The cell nucleus was stained with DAPI (4′,6-diamidino-2-phenylindole), shown in blue. (B) Electron microscopy of purified wt and K_259_A 2C^ATPase^ mutant viruses. Wild-type and mutant viruses were grown at 37°C and purified on cesium chloride density gradients (see Materials and Methods).

The inhibition of encapsidation, resulting in decreased amounts of infectious virus at 33°C (Fig. 6A), was supported by immunofluorescence imaging (Fig. 7A). PV 2C^ATPase^ K_259_A virus, grown at 37°C, was used to infect HeLa cells at 37, 35, and 33°C at an MOI of 5, and incubation continued for 4, 5, and 6 h, respectively, at the same temperatures. Infected cells were probed with monoclonal antibodies to 2C^ATPase^ and A12 antibodies, the latter recognizing mature virus (51). As shown in Fig. 7A, both the localization and estimated quantity of mature virus are comparable for the two viruses at 37 and 35°C. However, at 33°C there is a strong reduction in the amount of mature virus present in K_259_A-infected cells compared to the amount in cells infected with the wt virus. Notably, there are differences in the localization of 2C^ATPase^ in the wt and mutant virus-infected cells. In wt virus-infected cells, 2C^ATPase^ localizes in the perinuclear region of the cell, while with the mutant, this protein is primarily in the cytoplasm. Taken together, our results clearly indicate a relationship between the encapsidation defect of the K_259_A mutant at 33°C, the production of mature virus, and a delay in uncoating and protein synthesis during the next cycle of virus growth. It should be noted that an electron microscopic analysis of purified wt and K_259_A viral particles grown at 37°C did not reveal any detectable differences between the two viruses (Fig. 7B).

Our previous studies provided strong genetic evidence that in a CAV20-PV chimera (constructed of CAV20 P1 and PV P2 and -3 and designated CPP), the CAV20 VP3 “communicates” with PV 2C^ATPase^, an event required for assembly (1). We were able to support this assertion by coprecipitation experiments of *in vitro*-synthesized CAV20 VP3 and PV 2C^ATPase^ (1). Attempts to achieve similar results with PV VP3 and PV 2C^ATPase^ K_259_A have failed: we have observed coprecipitation of PV VP3 with both the wt and the mutant PV 2C^ATPase^ proteins (data not shown). We speculate that since the mutations in the viral 2C^ATPases^ of the polyproteins CPP and PPP (2C^ATPase^ N_252_S and K_259_A, respectively) map to very different positions in the proposed 2C^ATPase^ structure (hinge versus helix) (Fig. 2), they are differently exposed and available for potential interaction under the conditions of these experiments. On the other hand, *in vivo*, the interaction between capsid precursors and the replication complex involves very large, high-molecular-weight entities that may present the communicating residues in CPP or in PPP for binding in different ways.

## DISCUSSION

This study provides genetic evidence that a single amino acid replacement in a PV nonstructural protein (2C^ATPase^ K_259_A) generates variant virions with an unusual functional phenotype at 37°C: these virions that were assembled with wt proteins can infect cells at physiological temperature with wt efficiency, but they are defective in their ability to initiate infection at 33°C. We must conclude, therefore, that at 37°C the mutant nonstructural viral protein 2C^ATPase^ directed the formation of faulty PV virions.

Previous drug inhibition (9) and genetic experiments have led to the surprising observation that poliovirus 2C^ATPase^ was essential not only in genome replication but also in virion assembly (10–13). More surprising was the realization that an RNA packaging signal is not involved in poliovirus assembly (11), unlike with numerous other plus-strand RNA viruses (see, for example, references 52, 53, 54, and 55). Uniquely, the specificity of assembly results from an interaction of the nonstructural poliovirus protein 2C^ATPase^ with cognate capsid protein VP1 and/or VP3, or with capsid protein VP3 of C-cluster coxsackieviruses (10). Moreover, mutations influencing PV assembly seem to map to different parts of the 2C^ATPase^ polypeptide (Fig. 1C). A particularly powerful tool to deduce the role of PV 2C^ATPase^ in encapsidation was alanine scanning mutagenesis, implicating residues K_279_ and R_280_ and C_272_ and H_273_ within the C-terminal Zn^2+^ binding domain (residues 269 to 286) in encapsidation and/or uncoating (12, 13, 34). Suppressor variants of the K_279_A R_280_A mutant indicated that this site interacts with both capsid proteins VP1 and VP3, possibly in the context of one or more capsid precursors or the fully assembled capsid (12). On the other hand, suppressor variants of the C_272_A H_273_A mutant revealed an interaction with an upstream segment of 2C^ATPase^ that is located between boxes A and B of the NTP binding domain (12, 13). The K_259_ residue in poliovirus 2C^ATPase^ adds to the previously discovered locations near the C terminus of the polypeptide that are involved in encapsidation/uncoating. This domain extends from residue N_252_ to the M_293_ and K_295_ suppressor mutations of a linker insertion at residues 255 and 256 of the cold-sensitive mutant of Li and Baltimore (1).

Originally, the aim of our studies was to search for and identify one or more sites in protein 2C^ATPase^ near amino acid N_252_ that could be implicated in encapsidation. N_252_ was identified to be essential in the assembly of a CAV20/PV chimera constructed to contain the CAV20 capsid followed by poliovirus P2/P3 (10). N_252_ maps to a variable flexible region within PV 2C^ATPase^ (Fig. 2B to D), a site presumed suitable for an interaction between 2C^ATPase^ and a capsid polypeptide. To our surprise, we realized, however, that the asparagine at residue 252 of 2C^ATPase^ is not conserved among enteroviruses, not even between three poliovirus serotypes (Fig. 2E) or in CAV20, the capsid donor of the PV/CAV20 chimera. We speculated, therefore, that residues in the variable flexible region other than N_252_ or in addition to N_252_ might play a role as the presumed capsid-interacting site involved in particle assembly.

We first introduced triple alanine mutations into PV 2C^ATPase^ near N_252_ (Fig. 2B to D). We selected mutants for analysis that fell either into highly structured or flexible domains. We identified two triple alanine mutants (FMI/AAA and GKL/AAA) that possessed lethal growth phenotypes at all temperatures tested (33, 37, and 39.5°C). These mutations are predicted to be located in a β sheet and a helical domain, respectively, of the 2C^ATPase^ structural model. In addition, we observed two mutants that were *ts* and/or quasi-infectious and exhibited normal protein translation and processing profiles but were defective in RNA replication (EYS/AAA and QVM/AAA). These mutations fell into a partly or fully flexible stretch of residues in the predicted 2C^ATPase^ structure. Since the processes of encapsidation and RNA replication are linked, we cannot exclude the possibility that these mutants also had encapsidation defects, independent of RNA replication. It should be noted that an E_253_G change in PV 2C^ATPase^ was previously shown to yield a small-plaque virus, to prevent secretion inhibition in tissue culture cells (56), and to produce a valosin-containing protein (VCP)-knockdown-resistant PV mutant (38). Whether the E_253_A substitution, within the context of the EYS mutation, would cause similar defects is not yet known.

To identify the specific residues responsible for the lethal growth phenotypes of the triple alanine mutants, we scanned them by single alanine mutagenesis. The mutants included the F_244_A, I_248_A, G_258_A, K_259_A, and L_260_A residues that are highly conserved in picornavirus 2C^ATPase^ proteins and M_246_, which is less conserved (Fig. 2E). Mutant F_244_A was nonviable and had a severe defect in RNA replication (data not shown). Interestingly, our previous alanine mutagenesis of positively charged residues R_240_/R_241_ and D_245_/D_247_, in close vicinity to F_244_, also resulted in lethal growth phenotypes and severe replication defects (12). Three of the single alanine mutants (M_246_A, I_248_A, and L_260_A) were quasi-infectious and produced variants that had either wt or *ts* growth phenotypes. In all cases (M_246_A, I_246_A, and L_260_A), the variants contained an exchange of a moderately hydrophobic residue, alanine, with a more hydrophobic and larger amino acid, valine. The original residues, M_246_, I_246_, and L_260_, are also strongly hydrophobic and larger than the alanines that replaced them. It should also be noted that reversion to the original genotypes would have required two simultaneous nucleotide substitutions, while the A→V changes occurred with the replacement of a single nucleotide.

The last single alanine mutant, K_259_A, was cold sensitive and exhibited a delay in growth and in protein synthesis at the restrictive temperatures, 35°C and, particularly, 33°C. Our experiment with a reporter virus indicated a specific defect in encapsidation at these temperatures. Although this defect was not detectable at 37°C with this assay, an abnormality in virion structure, presumably resulting from imperfect encapsidation at this temperature, could be inferred from the observation that mutant viruses grown at 37°C were strongly delayed both in growth and in protein synthesis when used for infections and growth at 35 or 33°C (Fig. 5A and B). The 2C^ATPase^ K_259_A mutation alone did not confer such phenotypes onto a replicon (Fig. 6B), which pinpointed the defect to the virion structure of PV grown at 37°C. Immunofluorescence imaging of cells infected with the wt and mutant viruses confirmed the nearly total lack of mature virus production by the mutant at 33°C 6 h postinfection (Fig. 7). In addition, the imaging experiments revealed differences in the localization of 2C^ATPase^ with the wt and the mutant viruses at 33°C. While the wt virus exhibited 2C^ATPase^ localization in the perinuclear region of the cell, the mutant protein was primarily in the cytoplasm.

As mentioned above, the asparagine at position 252 is not conserved in an alignment of picornavirus 2C^ATPase^ proteins and even in different poliovirus serotypes (Fig. 2E). Not surprisingly, the N_252_ in PV1(M) can be replaced with A, G, S, or D without any deleterious effect on viral growth (10; unpublished data). Some residues in the immediate vicinity of N_252_ that were included in our mutational analyses (M_246_, Q_249_, M_251_, E_253_, and S_255_) are also poorly conserved (Fig. 2E). However, we suggest that the growth phenotypes of the mutants correlated with the extent of conservation of amino acids within this domain of the protein. Accordingly, the two mutants with the highest conservation (FMI/AAA, GKL/AAA) exhibited lethal phenotypes, while mutants (EYS/AAA and QVM/AAA) that were either *ts* or quasi-infectious, respectively, contained substitutions in the variable region.

With every replicative cycle, RNA viruses replicate their genomes with the astounding error rate of 10^−4^, and indeed, most RNA viruses have not developed any proofreading and editing functions. This phenomenon, for which RNA viruses are called quasispecies (57), is of great advantage for these infectious agents, but there is also a disadvantage: RNA viruses live under conditions of genetic austerity, e.g., their genome is very small. High error rates at every step of replication can be a burden, but as Kirkegaard realized first (14), PV has evolved to possess a powerful proofreading mechanism because of the stringent dependence of individual steps in replication in *cis* (12, 15, 38, 42, 58). In addition, Hogle has added that uncoating and encapsidation are linked since the normal release of the genome depends upon correctly assembled virion particles (20). The 2C^ATPase^ K_259_A mutant described here fits well into this proofreading scheme at the steps of encapsidation/uncoating.

Our studies reported here identify K_259_ as a residue critical for virion assembly and the subsequent step of uncoating during the next cycle of infection. Although many unanswered questions still exist about the roles of 2C^ATPase^ in morphogenesis and uncoating, these results together with those from our previous alanine scanning experiments demonstrate the usefulness of genetic analyses with *ts* and quasi-infectious variants for the identification of residues important for these processes.
